# Quality of Referral Letters Written by Family Physicians to Otologists -A Peer Assessment

**DOI:** 10.22038/ijorl.2019.35908.2187

**Published:** 2019-11

**Authors:** Mohammad Faramarzi, Mahmood Shishegar, Gholam Abbas Sabz, Sareh Roosta, Mehrdad Askarian

**Affiliations:** 1 Otolaryngology Research Center, Shiraz University of Medical Sciences, Shiraz, Iran.; 2 Otolaryngologist, Yasuj University of Medical Sciences, Yasuj, Iran.; 3 MSc of Biostatistics, Otolaryngology Research Center, Shiraz University of Medical Sciences, Shiraz, Iran.; 4 Department of Community Medicine, Medicinal and Natural Products Chemistry Research Center, Shiraz University of Medical Sciences, Shiraz, Iran.

**Keywords:** Family physicians, Otolaryngology, Otology

## Abstract

**Introduction:**

Otolaryngology is a field with a high referral rate; however, there is a dearth of research on the quality of referral letters written in this field. This study was carried out to explicitly assess the quality of referral letters, more specifically in the field of otology.

**Materials and Methods:**

Two otologists assessed referral letters written by general practitioners or primary care physicians working as family physicians. They were asked to make independent assessment on different variables related to the quality of referral letters and their appropriateness. A “qualified referral letter” in the current study is defined as a letter with standard items, including, description of chief complaint, description of associated symptoms, relevant physical findings, past medical history, drug history, family history, and reasons for referral.

**Results:**

A total of 1000 referral letters written by 652 primary care physicians were investigated in the current study. The obtained results indicated that 74% of referral letters to otologists contained inadequate information regarding various items in the referral letters. Symptoms, diagnosis, and signs were only reported in 28.3%, 28.9%, and 3.6% of the letters, respectively. The findings showed that most common reasons for referrals were uncertainty in diagnosis (52.4%), persistence of the patient (32.6%), and failed therapy (32%). With regards to case-specific conditions, the highest referral rates were related to external otitis, otitis media with effusion, and acute otitis media.

**Conclusion:**

According to the obtained results of the current study, the content of referral letters were insufficient or inappropriate. Therefore, it is recommended to improve otolaryngology syllabus and provide suitable courses for undergraduate students in order to become familiar with the importance of referral letter writing.

## Introduction

Health care quality is a global problem with substantial economic and political burden. The development and implementation of the family physician program in some countries, including Iran, have positively affected healthcare services, especially in rural areas (1). Due to the workload of specialists and expenses of healthcare services in Iran, the Ministry of Health and Medical Education of Iran similar to its counterparts in other countries has executed the family physician program, which acts as a gatekeeper. In this regard, there should be a qualified channel of connection between specialists and referral loop. 

Although there is an Academic residency program to become a family physician in Iran, most family physicians are general practitioners who act as primary care physicians (PCPs). Otolaryngology is a medical field with high referral rate, accounting for approximately 50% of child referrals (2). It has been shown that otolaryngology is the third or fifth most common specialty involving the referral process (3-5).Currently, referral letters are the most common means of communication between the PCPs and specialists worldwide. Despite the importance of the referral process, little is known about qualified referral letters in the field of otology. For instance, Dupont evaluated the quality of 600 referral letters from family physicians to dermatologists. He found that only less than 50% of the letters provided the necessary information about the treatments (11).

The essential elements of structural referral letters include reason for referral, medical history including important co-morbidities, clinical signs and symptoms, para-clinical examination results, current medication, and diagnosis (6). Although the main items of referral letters have been standardized by the Ministry of Health and Medical Education in Iran to enhance the quality of referral letters, it seems that the content of referral letters is substandard and unsatisfactory (7-10).

With this background in mind, the current study was motivated to conduct a peer assessment of the quality of referral letters as the means of communication between PCPs and otologists. In order words, the present study aimed to detect the problems in this type of letters. Therefore, the obtained results could be beneficial not only for healthcare providers but also for program directors in the field of medical education.

## Materials and Methods

This cross-sectional study was approved by the Local Institutional Review Board of Shiraz University of Medical Sciences (approval no. 6007), Shiraz, Iran. Shiraz is the largest city in the south of Iran with a population of 1.8 million. All referral letters that were included without any specific inclusion criteria for selection of letters. All of them were Witten by PCPs working as family physicians in Shiraz. All PCPs are medical university graduates from Iran. Two academic otologists independently visited the patients in Motahari Outpatient Clinic affiliated with Shiraz University of Medical Sciences. This clinic as a tertiary referral center is the largest outpatient university-affiliated clinic in south of Iran. Each of otologists were independently asked to make decisions on the 14 items in the checklist. The investigated variables related to the quality of referral letters included errors or inattentiveness in referral letters, current chief complaint, description of associated symptoms, relevant physical findings, past medical history, drug history, family history, reason for referral, appropriate para-clinical investigations and their results, as well as provisional diagnosis or clinical impression. Moreover, the otologists asked the investigated patients if the cause for referral was PCPs’ decision or patients’ persistence. In addition, there was a question asking otologists whether the referral letter contents contained sufficient data for decision making. Another questions dealt with otologists’ opinions about the reasons for referrals including uncertainty in PCPs’ diagnosis, failed therapy, and inaccessibility to specialized diagnostic tools or special tests (e.g., imaging investigation). Additional items in the checklist addressed the demographic characteristics of PCPs, such as age, gender, and years of practice. P-value less than 0.05 was considered statistically significant. Although the sample size was calculated at 382 referral letters in the current study, the researchers enhanced the research accuracy by analyzing 1000 referral letters. The reason for this was that a larger number of patients and referral letters were available at Motahari Outpatient Clinic of Shiraz University. The collected data during March 2017 to December 2017 were analyzed using SPSS software (version 15 for Windows; SPSS Inc., Chicago, III., USA) for descriptive statistics. The obtained results were reported as mean±SD or frequency and percentage. 

## Results

A total of 1000 referral letters in the current study were written by 652 PCPs. The mean age of PCPs was 32.6 years (range: 25-57 years). Regarding gender distribution, 58% of PCPs were male. The mean duration of their practices was 12 years (range: 4-396 months). The referral letters were for 606 female (60.6%) and 394 male (39.4%) patients with the age range of 8 months to 82 years (mean±SD: 40.03±16.76 years).The errors and inattentiveness of PCPs regarding the past medical history and drug history of patients were observed in 354 (35.4%) and 396 (39.6%) of referral letters. Out of 280 (28%) illegible or poorly written letters, 170 (60.7%) and 110 (39.3%) ones were English and Persian, respectively. The findings also showed that 740 (74%) of referral letters contained insufficient amount of data in order to make an appropriate decision. Detected signs were only written in 36 letters (3.6%) out of which 19 (52.8%) were written correctly. The most common signs in referral letters were otorrhea, inflammation of external ear canal, and tympanic membrane perforation (Table.1).

**Table 1:** Documented signs in the investigated referral letters

***Signs***	***N***	***%***
*Otorrhea*	12	63.2
*Inflammation of External Canal*	9	47.4
*Tympanic Membrane Perforation*	8	42.1
*Congested Tympanic Membrane*	6	31.6
*Fungal Infection*	5	26.3
*Compact Wax*	5	26.3
*Facial Nerve Paralysis*	2	10.5
*Tympanosclerotic Plaque*	1	5.3
*Middle Ear Cholesteatoma*	1	5.3
*Middle Ear Granulation tissue*	1	5.3

Note: Total percentage is more than 100% due to multiple reasons for referral.

Symptoms were indicated in 283 (28.3%) letters out of which 58 (20.5%) were correctly reported. The most common symptoms were hearing loss, earache, and ear secretion (Table.2). 

**Table 2:** Documented symptoms in the investigated referral letters

***Symptoms***	***N***	***%***
*Hearing Loss*	21	36.2
*Pain*	14	24.1
*Ear Secretion*	13	22.4
*Itching*	11	19.0
*Dizziness*	9	15.5
*Tinnitus*	4	6.9
*Facial Asymmetry*	2	3.4

Note: Total percentage is more than 100%, due to multiple reasons for referral.

As can be seen in Table 3, the most common reasons for referrals were related to the uncertainty in diagnosis (52.4%), patients’ persistent (32.6%), and failed therapy (32%).

**Table 3:** Reasons for referral in the investigated referral letters

***Reasons***	***N***	***%***
*Uncertainty in Diagnosis*	524	52.4
*Patients' persistent*	326	32.6
*Failed Therapy*	320	32.0
*Advice for Management*	287	28.7
*Requiring Special Tests or Services*	169	16.9

Note: Total percentage is more than 100% due to multiple reasons for referral

The analysis of 1000 referral letters indicated that diagnosis were written in 289 (28.9%) letters out of which 51 (17.6%) were right diagnoses. As presented in Table 4, otologists considered external otitis, otitis media with effusion, and acute otitis media as the three most common ear problems (14.5%, 12.8%, and 11.7%, respectively).

**Table 4:** Common referred conditions in the investigated referral letters

***Diagnosis ***	***N***	***%***
*External Otitis*	145	14.5
*Otitis Media with Effusion*	128	12.8
*Acute Otitis Media*	117	11.7
*Sensory Neural Hearing Loss*	114	11.4
*Inactive Chronic Otitis Media*	101	10.1
*Normal*	92	9.2
*Active Chronic Otitis Media without Cholesteatoma*	83	8.3
*External Canal Wax*	76	7.6
*Benign Positional Vertigo*	58	5.8
*Active Chronic Otitis Media with Cholesteatoma*	34	3.4
*Otosclerosis*	31	3.1
*Menier's Disease*	18	1.8
*Bell's Palsy*	2	0.2
*Glomus Tumor*	1	0.1

## Discussion

Currently, referral letter itself is a principal means through which an effective professional relationship is established between PCPs and specialists. The content of referral letters is the pivotal aspect of referral process. The findings of the current study indicated that 74% of referral letters contained insufficient data for decision making. In the same vein, many studies mostly in western countries have revealed that referral letters do not convey relevant and adequate information. For example, in a study performed by Jenkins on the quality of general practitioner referrals, it was indicated that errors and omissions regarding past medical history and drug history were in 28% and 26% of referral letters (9). In a study conducted by Forrest et al., the analysis of referrals by 122 pediatricians revealed that no information was transferred to the specialist in 49% of cases (7). In another study, Forrest et al. concluded that family physicians reported only a sign or symptom for one in five referrals (3). In the present study, the obtained results indicated that the 3.6% and 28.3% of referral letters contained information about signs and symptoms, respectively. Moreover, it was found that only 52.7% and 20.5% of the reported signs and symptoms were correct, respectively. In another study conducted by Dupont on the quality of 600 referral letters from family physicians to dermatologists, he mentioned that less than 50% of letters conveyed information about previous treatments (11). 

In the present study, the most common reasons for referrals were related to uncertainty in diagnosis (52.4%), persistence of the patient (32.6%), failed therapy (32%), advice seeking for disease management (28.7%), and inaccessibility to specialized diagnostic tools or special tests, such as imaging investigation (16.9%). In addition, the three most common documented symptoms in referral letters were hearing loss, otalgia, and otorrhea. 

Regarding case-specific conditions, the highest referral rates were accounted for external otitis, otitis media with effusion, and acute otitis media. Moreover, uncertainty in diagnosis was the most common cause of referrals to otolaryngologists in the current study.

It is commonly believed that PCPs can manage most patients suffering from earache or diagnose the common causes of hearing loss in their community. However, it is worth mentioning that some therapeutic or surgical interventions (e.g., sensory neural hearing loss, management of chronic otitis media with cholesteatoma, complicated chronic otitis media) are beyond the realm of PCP’s knowledge and skills. Some studies showed that family physicians can manage 95% of patients in outpatient centers (3). However, a study of 100 patients suffering from earache by Worrall indicated that rural family physicians could not diagnose the causes of earache in 94 cases (16). It was reported that parents whose children suffered from recurrent acute otitis media or otitis media with effusion affect the decisions made by family physicians. Moreover, family physicians appear to have lower thresholds than pediatricians for the referral of those patients (17). It is generally confirmed that the PCPs’ referral thresholds are based on their values, clinical abilities, and experiences (18). Therefore, the obtained results of the current study highlights the importance of community-based education and mastery of otolaryngology in a properly designed course during undergraduate period. In addition, it has been suggested that both PCPs and consultants develop a program by the integration of clinical guidelines and educational curriculum in order to effectively process the referrals (19-21).

On the other hand, the second most common cause of referral was the persistence of patients, which counts for about one-third of referrals. Similar to other studies, the findings of the current study revealed that the common causes of high referral rate or seemingly inappropriate referrals are patients’ expectation and their insistence (22,23). This rate was reported at 12.2-56% for different regions of Iran (24-26). In a study conducted by Jenkins on 705 referrals, he noted that 13% of the referrals were inappropriate, which this rate was significantly higher in medical specialties than the referral rate to surgeons (19.6% versus 8.6%,( 9).

According to Fertig et al., specialists believe that 9.6% of all referrals by general practitioners are inappropriate. Also otolaryngologists considered that 1.11% of them are inappropriate (27). However, in some countries including Iran, the Ministry of Health has recommended that a 10% referral rate is reasonable (28,29). However, some PCPs believe that their strict roles can act as gatekeepers and refusal of referrals can have detrimental effect on the physician-patient relationship (30). On the other hand, a high rate of referrals can result in a higher level of satisfaction among patients (31). As a fundamental issue in medical professionalism, physicians should be concerned about the cultural dimension of their patients’ needs and expectations (32). On the other hand, paternalistic clinical setting is no longer acceptable, and it is essential to follow a patient-centered care approach (33).

With this background in mind, it is important to ask if the high rate of referral is the main problem in the referral loop. High referral rate is sometimes perceived as an inappropriate issue, whereas it is highly appropriate since it reassures PCPs and patients. However, this threshold of reassurance can vary among PCPs and patients (34). Barnett et al. identified during 1999-2009 there was a 94% surge of ambulatory referrals from 4.8% to 9.3% (35). In addition, over two-thirds of patients (68%) who were visited by a specialist were referred by a family physician (6). In the United States, more than a third of patients are annually referred to specialist for consultation, and specialist visits are more than half of outpatient visits (36). A review of literature showed that the referral rate to specialists was approximately 4.5-20.0%(3,4,28,29,35,37-39). In fact, the number of referral rate itself is not an appropriate indicator of the quality and effectiveness of the referral process (27,40-42). In other words, the effectiveness of a referral system should be assessed based on its outcomes. Bertakis et al. mentioned that older patients, severity of physical condition, and a significant number of previous visits by PCPs were referral predictive factors to specialists (43). On the other hand, under-referral is the main problem rather than over-referral. It has been shown that 26% of malpractice assertions are related to an overlooked or delayed diagnosis as a result of restrictive policy on referrals by PCPs (41,42). Accordingly, it has been suggested that it is not appropriate to impose severe restrictions on referrals to specialists (31). 

It should be noted that the current research may have some limitations. At first, it focused on a single clinical field of otology, which constitutes a limited source of information. In order to produce a general concept, it is essential to study other fields of medicine, as well. Moreover, the obtained data regarding patients’ persistent rate could be biased since that data were collected from PCPs’ point of view not based on patients’ clear and original conception. 

## Conclusion

One of the major findings of the current study was related to the most common diseases a PCPs may encounter in the field of otology. The obtained results of the current study confirmed that the written referral letters by PCPs were poorly written and contained insufficient and inappropriate information in the field of otology. In this regard, it is suggested that program directors design and conduct a proper writing course in medical education. Finally, in order to improve the quality of the referral process, further studies should be carried out to address PCPs’ opinion about the feedback letters from specialists in the referral loop. Generally, these issues in referral process seem to be essential requirements to enhance health care quality as a global concern. 
